# Exploring difficult-to-manage axial spondyloarthritis: results from a Dutch clinical practice registry

**DOI:** 10.1093/rheumatology/keaf120

**Published:** 2025-02-28

**Authors:** Marius L Smits, Casper Webers, Harald E Vonkeman, Astrid van Tubergen

**Affiliations:** Department of Rheumatology, Maastricht University Medical Centre+, Maastricht, The Netherlands; Care and Public Health Research Institute (CAPHRI), Maastricht University, Maastricht, The Netherlands; Department of Rheumatology, Maastricht University Medical Centre+, Maastricht, The Netherlands; Care and Public Health Research Institute (CAPHRI), Maastricht University, Maastricht, The Netherlands; Department of Rheumatology and Clinical Immunology, Medisch Spectrum Twente, Enschede, The Netherlands; Department of Psychology, Health and Technology, University of Twente, Enschede, The Netherlands; Department of Rheumatology, Maastricht University Medical Centre+, Maastricht, The Netherlands; Care and Public Health Research Institute (CAPHRI), Maastricht University, Maastricht, The Netherlands

**Keywords:** axial spondyloarthritis, difficult-to-manage, epidemiology, prevalence, risk factors

## Abstract

**Objectives:**

To explore (1) the prevalence of difficult-to-manage axial spondyloarthritis (D2M axSpA) in clinical practice according to the Assessment of SpondyloArthritis international Society (ASAS) definition, (2) specific components of this definition contributing most to its fulfilment and (3) associated patient characteristics.

**Methods:**

This cross-sectional study used data from the SpA-Net registry. Fulfilling the ASAS definition required: (1) treatment failure, (2) insufficient disease control (any of: [A] high disease activity (AxSpA Disease Activity Score [ASDAS] ≥2.1), [B] signs of active disease (e.g. peripheral manifestations) or [C] reduced health-related quality of life) and (3) a problematic management situation in the patient’s or rheumatologist’s perspective. Three variations of the ASAS definition were investigated, including only subcriterion A, B or C in domain 2. Treatment-refractory axSpA required fulfilment of the primary ASAS definition, an ASDAS ≥2.1 and CRP ≥5 mg/L. The prevalence of D2M axSpA per explored definition was calculated, and associated characteristics were assessed using logistic regression analyses.

**Results:**

Data from 263 patients were analysed. Overall, 9.7% had D2M axSpA (variations A–C: 3.6–8.7%) and 1.7% had treatment-refractory disease. ‘Treatment failure’ affected 9.9–11.6% of patients, ‘insufficient disease control’ affected 79.7% of patients (variation A: 57.0%, B: 44.6%, C: 51.4%) and ‘problematic management’ affected 51.4–60.2% of patients. Current smoking (OR = 3.1 [95% CI 1.1–8.7]) and a history of psoriasis (OR = 2.8 [95% CI 1.0–7.6]) were associated characteristics.

**Conclusion:**

One in 10 patients with axSpA have D2M disease. Patient-reported outcomes contribute importantly to the classification of D2M axSpA. Current smoking and a history of psoriasis are associated characteristics.

Rheumatology key messages

- One in 10 patients with axial spondyloarthritis (axSpA) have difficult-to-manage (D2M) disease by the Assessment of SpondyloArthritis international Society (ASAS) definition, and 2% have treatment-refractory disease.
- Patient-reported outcomes contribute importantly to the classification of D2M axSpA, whereas treatment failure is the most prominent limiting factor to fulfil the ASAS definition.
- Current smoking and a history of psoriasis are associated with having D2M axSpA.

## Introduction

In managing axial spondyloarthritis (axSpA), remission of symptoms, maintenance of physical and psychosocial functioning and the prevention of complications are strived for [1]. The ongoing expansion of the treatment armamentarium, particularly the biological and targeted synthetic DMARDs (b/tsDMARDs), has contributed significantly to the feasibility of attaining these targets [2–4]. In addition, the development of instruments such as the Axial Spondyloarthritis Disease Activity Score (ASDAS) has facilitated consistent evaluations of treatment outcomes [1, 5].

Managing axSpA can be complex, however, in part due to its high heterogeneity [1]. In clinical practice, up to 40% of patients with axSpA may experience inadequate response to multiple anti-rheumatic therapies [6]. Furthermore, only 25% of patients with recent onset axSpA achieve remission within five years [7]. A subset of these patients, who additionally present with a disease management situation deemed problematic, may be considered to have ‘difficult-to-manage’ (D2M) axSpA [8]. The D2M construct encompasses a wide range of interplaying biological and contextual factors, which extend beyond pure ineffectiveness of treatment [9]. Although patients who exhibit objectively persistent inflammatory activity resulting from the intrinsic failure of multiple DMARDs (i.e. treatment-refractory [TR] axSpA) are part of the D2M construct, this group likely only represents a small portion of the D2M axSpA population [10].

The D2M phenotype has previously been described in other inflammatory rheumatic and musculoskeletal diseases (RMDs) under the term ‘difficult-to-treat’ (D2T), most prominently in RA and PsA [11–13]. D2M disease in RMDs has been associated with impaired function and participation for patients, and frustration and insecurity amongst healthcare providers [14]. Furthermore, higher societal costs due to work productivity loss and increased use of healthcare resources have been reported [14, 15]. A knowledge gap exists, however, regarding the prevalence of D2M axSpA and associated patient characteristics. Improved knowledge of this may help rheumatologists to identify and adequately manage patients with D2M axSpA [6].

Recently, a consensus definition for D2M axSpA was established by the Assessment of SpondyloArthritis international Society (ASAS), which requires fulfilment of three domains: (1) treatment failure, (2) insufficient control of signs and symptoms and (3) a problematic management situation from the perspective of the patient and/or rheumatologist [16]. The next step would be to investigate the implications of applying this definition in real-world patient populations. The first objective of this study was, therefore, to estimate the prevalence of D2M and TR axSpA according to the ASAS definition in a multicentre clinical practice SpA registry. Secondly, we aimed to explore which components of the ASAS definition most frequently contributed to being classified as having D2M axSpA by exploring different variations of this definition. Thirdly, we sought to identify which patient characteristics are associated with having D2M axSpA.

## Methods

### Study population and design

This cross-sectional study used data from SpA-Net, a disease-specific web-based health registry for patients with SpA (ICTRP Registration: NTR6740) [17]. All patients with a clinical diagnosis of SpA, and who were under rheumatology care in one of the two participating centres in the Netherlands (Maastricht University Medical Centre+ and Medisch Spectrum Twente), were consecutively included in SpA-Net as part of their routine care. Both prevalent and incident cases were included, and no other inclusion or exclusion criteria were applied. All patients were monitored and treated according to current management recommendations.

For the present study, patients with a clinical diagnosis of axSpA who had data recorded in SpA-Net between 1 January 2019 and 30 June 2023 were eligible if a treatment history and ≥1 ASDAS measurement were available. While SpA-Net was initiated in 2016, only observations entered from 2019 onwards were used to make certain that included measurements took place during a period in which sufficient treatment options with different modes of action (MoA, i.e. TNF-α, IL-17 and Janus kinase [JAK] inhibitors) were available. Strictly data from patients with a time since diagnosis of ≥1 year were included, ensuring each patient had sufficient opportunity to attempt multiple treatments. If data from multiple time points were available for a patient, the most recent complete entry was selected.

The Medical Research Ethics Committee of the Academic Hospital Maastricht/Maastricht University (METC azM/UM 15–4-266) determined that this study was not subject to the Dutch Medical Research Involving Human Subjects Act, considering that data from SpA-Net were collected as part of routine care. All patients provided written informed consent for the use of their data.

### Outcomes

**Figure 1.**
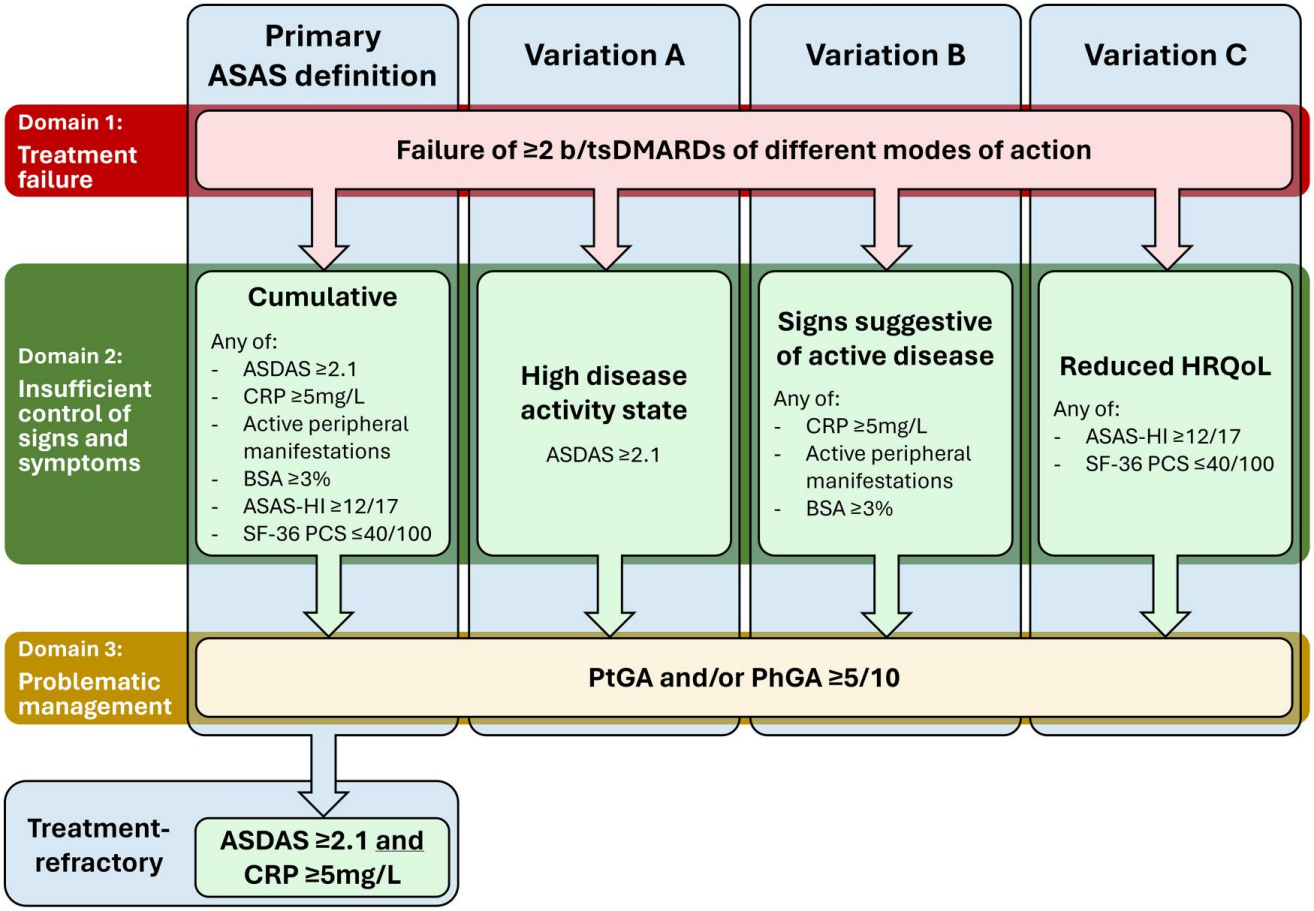
Primary ASAS definition of D2M axSpA and the three variations explored in this study. Patients were classified as having D2M axSpA if all three domains were fulfilled. Fulfilment of the primary ASAS definition with the presence of an ASDAS ≥2.1 and CRP ≥5 mg/L was required to be classified as treatment-refractory. Peripheral manifestations included arthritis, enthesitis and dactylitis. ASAS: Assessment of SpondyloArthritis international Society; ASAS-HI: ASAS Health Index; ASDAS: Axial Spondyloarthritis Disease Activity Score; axSpA: axial spondyloarthritis; bDMARD: biological DMARD; BSA: psoriasis body surface area; D2M: difficult-to-manage; HRQoL: health-related quality of life; PhGA: physician global assessment; PtGA: patient global assessment; SF-36 PCS: 36-Item Short Form Health Survey Physical Component Summary; tsDMARD: targeted synthetic DMARD

This study applied the ASAS definition of D2M axSpA (Fig. 1: ‘Primary ASAS definition’) [16], requiring patients to fulfil three domains adapted for data availability in SpA-Net:

Domain 1, ‘treatment failure’, was defined as treatment according to ASAS-EULAR recommendations and failure of ≥2 b/tsDMARDs of different MoA, attributed to side effects or a lack or loss of treatment response [1].

Domain 2, ‘insufficient control of signs and symptoms’, was defined as the presence of ≥1 of the following three subcriteria: (A) a *high disease activity state* (ASDAS ≥2.1) [18], (B) *signs suggestive of active disease*, including a CRP ≥5 mg/L, active peripheral manifestations (arthritis, enthesitis or dactylitis) and/or a psoriasis body surface area (BSA) ≥3% [19], or (C) a *reduced health-related quality of life (HRQoL)*, measured by an ASAS Health Index (ASAS-HI) ≥12/17 and/or 36-Item Short Form Health Survey Physical Component Summary (SF-36 PCS) ≤40/100 [20, 21].

Domain 3, ‘problematic management’, was defined as a patient and/or physician global assessment (PtGA/PhGA) ≥5/10. These instruments and cut-off value were chosen in the absence of a specific measurement instrument in SpA-Net for this domain, and in accordance with previous research on D2T RA [22, 23].

Additionally, TR axSpA was defined as fulfilment of all three domains, with both an ASDAS ≥2.1 and CRP ≥5 mg/L [16].

Our main interest was the prevalence of D2M and TR axSpA by the primary ASAS definition. As a secondary outcome, we investigated three variations of this definition to explore how specific components included in domain 2 contributed to being classified as having D2M axSpA (Fig. 1: ‘Variations A–C’).

### Covariables

Additional sociodemographic and disease characteristics of patients collected from SpA-Net were sex, age, education (attainment of university or higher professional education *vs* lower education), employment status (having paid work *vs* no paid work), smoking status (current smoker *vs* previous or non-smoker), symptom duration, diagnostic delay, HLA-B27 positivity, history of extra-musculoskeletal manifestations (EMMs; psoriasis, IBD and uveitis) and history of peripheral manifestations. Other characteristics and outcomes of patients collected were the current treatment(s) used (NSAIDs, conventional synthetic DMARDs, bDMARDs, tsDMARDs and systemic glucocorticoids), BASDAI [24], back pain on a 0–10 visual analogue scale (VAS) and fulfilment of the patient/physician acceptable symptom state (PASS-patient/PASS-physician) [25].

### Statistical analysis

Firstly, the demographic, disease and treatment characteristics of included patients were described, and the frequencies of patients fulfilling (each domain of) the primary ASAS definition of D2M and TR axSpA were determined. This was repeated for the three exploratory variations of the ASAS definition. Secondly, the characteristics of the patients belonging to the D2M+/D2M− groups were compared using independent samples *t*-tests for continuous variables, and *χ*^2^ or Fisher’s exact tests for categorical variables, as appropriate. Finally, univariable and multivariable logistic regression analyses were conducted to identify patient characteristics associated with fulfilling the primary ASAS definition. The multivariable models, adjusted for age and sex, included the demographic and disease characteristics that were potentially associated with D2M axSpA (*P* < 0.20) in the univariable analyses. Missing data were not imputed. The analysis of each definition included patients with available data on any of the instruments considered in each domain.

Three sensitivity analyses were conducted. Firstly, only patients with complete data for every instrument considered per definition were included in the respective analyses (‘completer analysis’). Secondly, the prevalence of D2M and TR axSpA was assessed using a lower cut-off value for the PtGA/PhGA in the ‘problematic management’ domain of ≥4/10 instead of ≥5/10 (in accordance with a proposed recommendation [26]). Thirdly, the prevalence of D2M and TR axSpA was calculated using the PASS-patient/PASS-physician instead of the PtGA/PhGA to assess the ‘problematic management’ domain. The PASS was not used in the main analysis as it was only added to SpA-Net in June 2020, several years after SpA-Net’s launch.

*P*-values of <0.05 were considered statistically significant. All analyses were conducted in IBM SPSS Statistics, Version 28.0 (IBM Corp., Armonk, NY, United States).

## Results

### Population characteristics

A b/tsDMARD history and ≥1 ASDAS measurement were available for 323 patients with a time since diagnosis of ≥1 year, of which 263 additionally had data available on PtGA/PhGA measurements (Supplementary Fig. S1, available at *Rheumatology* online). Within this base population of 263 patients, 120 (45.6%) were female, the mean age was 52.1 (s.d. 13.8) years, the mean symptom duration was 21.7 (s.d. 12.7) years, the mean ASDAS was 2.4 (s.d. 1.0) and 134 (51.0%) patients had a history of using ≥1 b/tsDMARD (Table 1).

**Table 1.** Characteristics of patients in the base population (*n* = 263)

Variable	Value
Female sex, *n* (%)	120 (45.6)
Age, years	52.1 (13.8)
Higher education, *n* (%)	73 (36.1)
No paid work, *n* (%)	91 (44.2)
Current smoking, *n* (%)	44 (21.7)
Symptom duration, years	21.7 (12.7)
Diagnostic delay, years	7.0 (9.0)
HLA-B27 positive, *n* (%)	166 (71.9)
History of EMMs	
Psoriasis, *n* (%)	44 (16.9)
IBD, *n* (%)	30 (11.5)
Uveitis, *n* (%)	60 (23.1)
History of peripheral manifestations^a^, *n* (%)	113 (43.5)
Current medication use	
None, *n* (%)	19 (7.2)
NSAID, *n* (%)	173 (65.8)
csDMARD, *n* (%)	29 (11.0)
bDMARD: TNFi, *n* (%)	123 (46.8)
bDMARD: IL-17i, *n* (%)	27 (10.3)
bDMARD: other, *n* (%)	5 (1.9)
tsDMARD, *n* (%)	5 (1.9)
Systemic glucocorticoid, *n* (%)	4 (1.5)
ASDAS	2.4 (1.0)
BASDAI, 0–10	4.5 (2.4)
CRP, mg/L	4.9 (7.6)
CRP ≥5mg/L, *n* (%)	77 (29.3)
PtGA, 0–10	4.7 (2.8)
Back pain VAS, 0–10	5.0 (2.8)
ASAS-HI, 0–17	5.8 (3.9)
SF-36 PCS, 0–100	40.6 (9.4)
SF-36 MCS, 0–100	48.4 (10.8)
PASS-patient, *n* (%)	94 (67.6)
PhGA, 0–10	2.0 (1.7)
PASS-physician, *n* (%)	108 (88.5)
Active peripheral manifestations^a^, *n* (%)	31 (15.4)
BSA ≥3%, *n* (%)	1 (0.5)

All values presented as mean (s.d.), unless otherwise indicated.

Number of patients with a missing value: higher education (*n* = 61), no paid work (*n* = 57), current smoking (*n* = 60), symptom duration (*n* = 42), diagnostic delay (*n* = 42), HLA-B27 (*n* = 32), history of EMMs (*n* = 3), history of peripheral manifestations (*n* = 3), ASAS-HI (*n* = 92), SF-36 (*n* = 77), PASS-patient (*n* = 124), PhGA (*n* = 47), PASS-physician (*n* = 141), active peripheral manifestations (*n* = 62), BSA (*n* = 76).

a Peripheral manifestations included arthritis, enthesitis and dactylitis.

ASAS: Assessment of SpondyloArthritis international Society; ASAS-HI: ASAS Health Index; ASDAS: Axial Spondyloarthritis Disease Activity Score; bDMARD: biological DMARD; BSA: psoriasis body surface area; csDMARD: conventional synthetic DMARD; D2M: difficult-to-manage; EMM: extra-musculoskeletal manifestation; IL-17i: IL-17 inhibitor; PASS: patient/physician acceptable symptom state; PhGA: physician global assessment; PtGA: patient global assessment; SF-36 MCS: 36-Item Short Form Health Survey Mental Component Summary; SF-36 PCS: SF-36 Physical Component Summary; TNFi: TNF inhibitor; tsDMARD: targeted synthetic DMARD; VAS: visual analogue scale.

Within the base population, 236 of 263 patients had data available on the instruments considered in the ‘insufficient control of signs and symptoms’ domain of the primary ASAS definition. Regarding the three exploratory variations, all 263 patients had data on the ASDAS (variation A), 222 patients had data available pertaining to signs suggestive of active disease (variation B) and 181 patients had data available on HRQoL instruments (variation C). Patient characteristics were comparable across the subpopulations used to assess each explored variation of the ASAS definition (Supplementary Table S1, available at *Rheumatology* online).

### Prevalence of D2M axSpA

**Figure 2.**
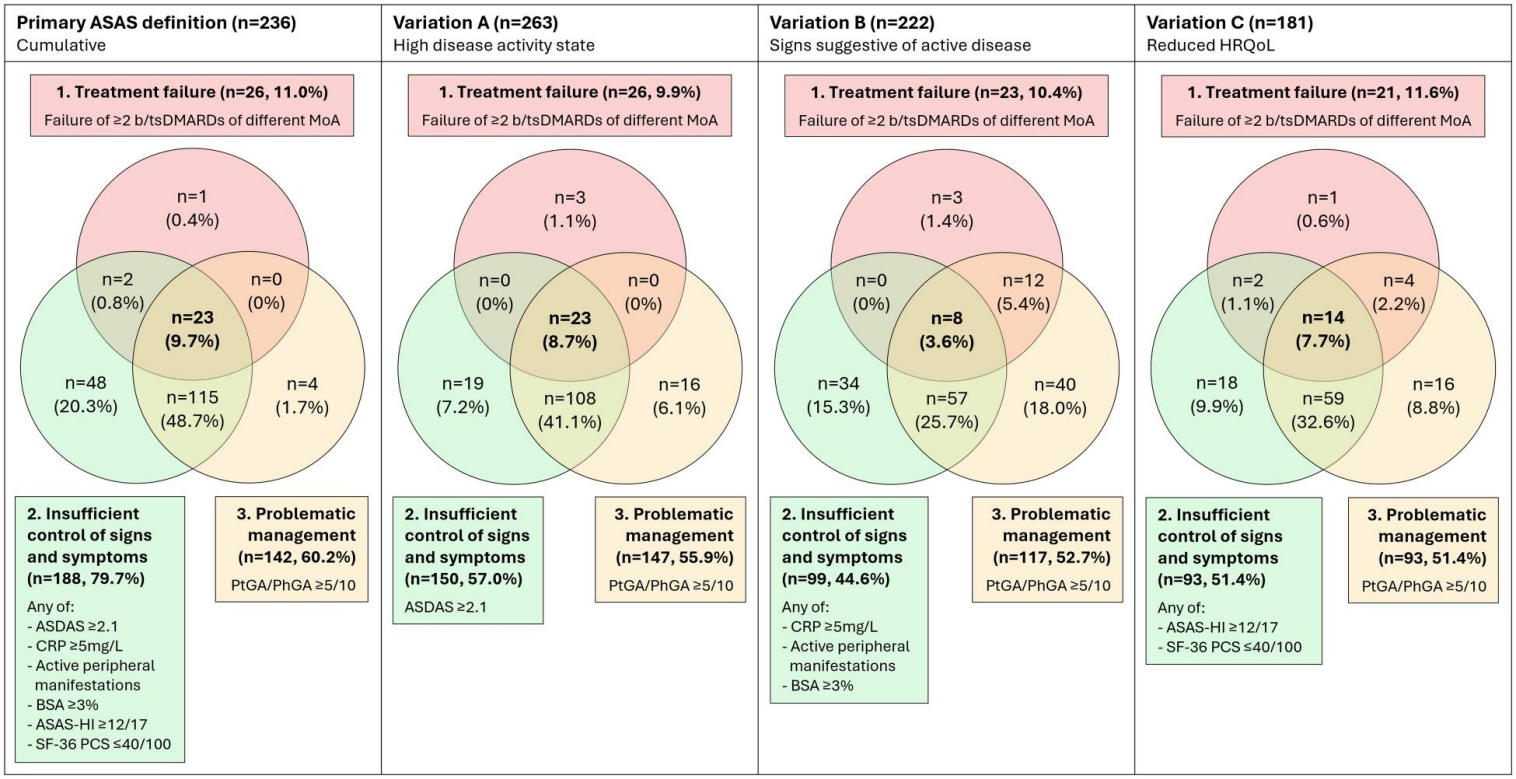
Percentages of patients fulfilling the primary ASAS definition and three exploratory variations, as well as the individual domains within the definitions. Percentages of patients fulfilling each individual domain per definition are presented in the respective boxes. For example, 26 of 236 (11.0%) patients fulfilled the ‘treatment failure’ domain in total in the population considered in the primary ASAS definition, irrespective of the other two domains. The analysis of each definition included patients with available data on any of the instruments considered in each domain. Peripheral manifestations included arthritis, enthesitis and dactylitis. ASAS: Assessment of SpondyloArthritis international Society; ASAS-HI: ASAS Health Index; ASDAS: Axial Spondyloarthritis Disease Activity Score; bDMARD: biological DMARD; BSA: psoriasis body surface area; HRQoL: health-related quality of life; MoA: mode of action; PhGA: physician global assessment; PtGA: patient global assessment; SF-36 PCS: 36-Item Short Form Health Survey Physical Component Summary; tsDMARD: targeted synthetic DMARD

Figure 2 presents the percentages of patients fulfilling (each domain of) the ASAS definition. The primary ASAS definition of D2M axSpA was fulfilled by 23 of 236 (9.7%) patients, of which four (1.7%) were additionally considered to have TR disease. In the three variations explored, 23 of 263 (8.7%) patients were classified as having D2M axSpA when only the subcriterion of high ASDAS-based disease activity was applied in the ‘insufficient control of signs and symptoms’ domain (variation A), eight of 222 (3.6%) when only signs suggestive of active disease were considered (variation B) and 14 of 181 (7.7%) patients when only a reduced HRQoL was used (variation C).

Regarding the individual domains, ‘treatment failure’ was experienced by 9.9–11.6% of patients. The ‘insufficient control of signs and symptoms’ domain was fulfilled by 79.7% of patients in the primary ASAS definition, 57.0% in variation A, 44.6% in variation B and 51.4% in variation C. The ‘problematic management’ domain was fulfilled by 51.4–60.2% of patients. Further exploration of the PtGA/PhGA scores used to define the ‘problematic management’ domain revealed that 49.7–58.9% of patients had a PtGA ≥5/10, 8.6–9.4% had a PhGA ≥5/10 and only 6.8–7.9% had both a PtGA and PhGA ≥5/10. Many patients fulfilled more than one domain. Notably, while 25.7–48.7% of patients fulfilled both the ‘insufficient control of signs and symptoms’ and ‘problematic management’ domains, only a minority of these patients also experienced ‘treatment failure’ and were classified as having D2M axSpA.

### Characteristics associated with D2M axSpA

Female sex, having no paid work, current smoking and a history of psoriasis, uveitis or peripheral manifestations were more frequently observed in the D2M+ groups compared with the D2M− groups, however, statistical significance of between-group differences varied between definitions (Table 2). In the multivariable analysis, current smoking (OR = 3.1 [95% CI 1.1–8.7]) and a history of psoriasis (OR = 2.8 [95% CI 1.0–7.6]) were associated with having D2M axSpA (Table 3).

**Table 2.** Demographic and disease characteristics of patients in the D2M and non-D2M groups per definition

Variable	Primary ASAS definition (cumulative^a^) *n* = 236			Variation A (high disease activity) *n* = 263			Variation B (signs of active disease) *n* = 222			Variation C (reduced HRQoL) *n* = 181		
	D2M+ *n* = 23	D2M− *n* = 213	*P*	D2M+ *n* = 23	D2M− *n* = 240	*P*	D2M+ *n* = 8	D2M− *n* = 214	*P*	D2M+ *n* = 14	D2M− *n* = 167	*P*
Female sex, *n* (%)	14 (60.9)	95 (44.6)	0.14	14 (60.9)	106 (44.2)	0.12	5 (62.5)	94 (43.9)	0.47	10 (71.4)	75 (44.9)	0.06
Age, years	51.6 (12.8)	52.1 (13.9)	0.85	51.6 (12.8)	52.2 (13.9)	0.84	54.9 (10.1)	52.1 (13.9)	0.59	52.2 (13.6)	51.7 (13.4)	0.90
Higher education, *n* (%)	8 (38.1)	61 (36.7)	0.90	8 (38.1)	65 (35.9)	0.84	2 (28.6)	66 (37.7)	1.00	7 (50.0)	59 (36.9)	0.33
No paid work, *n* (%)	12 (57.1)	75 (44.1)	0.26	12 (57.1)	79 (42.7)	0.21	5 (71.4)	81 (45.3)	0.25	9 (64.3)	69 (42.1)	0.11
Current smoking, *n* (%)	8 (38.1)	34 (20.4)	0.09	8 (38.1)	36 (19.8)	0.09	4 (57.1)	34 (19.3)	0.03	4 (28.6)	30 (18.5)	0.48
Symptom duration, years	22.4 (15.1)	21.3 (12.5)	0.72	22.4 (15.1)	21.6 (12.4)	0.80	18.7 (11.4)	21.9 (12.6)	0.54	22.8 (17.9)	21.0 (12.1)	0.73
Diagnostic delay, years	8.3 (12.8)	6.7 (8.3)	0.57	8.3 (12.8)	6.9 (8.6)	0.61	4.4 (3.0)	7.2 (9.3)	0.47	10.2 (15.4)	6.1 (7.5)	0.36
HLA-B27 positive, *n* (%)	14 (63.6)	137 (73.3)	0.34	14 (63.6)	152 (72.7)	0.37	5 (62.5)	131 (70.1)	0.70	8 (61.5)	105 (70.5)	0.54
History of EMMs												
Psoriasis, *n* (%)	8 (36.4)	34 (16.1)	0.04	8 (36.4)	36 (15.1)	0.02	3 (37.5)	38 (17.9)	0.17	7 (53.8)	30 (18.0)	0.01
IBD, *n* (%)	1 (4.5)	28 (13.3)	0.33	1 (4.5)	29 (12.2)	0.49	1 (12.5)	25 (11.8)	1.00	1 (7.7)	22 (13.2)	1.00
Uveitis, *n* (%)	8 (36.4)	49 (23.2)	0.17	8 (36.4)	52 (21.8)	0.12	3 (37.5)	45 (21.2)	0.38	5 (38.5)	38 (22.8)	0.31
History of peripheral manifestations^b^, *n* (%)	12 (54.5)	93 (44.1)	0.35	12 (54.5)	101 (42.4)	0.27	5 (62.5)	99 (46.7)	0.48	10 (76.9)	84 (50.3)	0.06

All values presented as mean (s.d.) unless otherwise indicated. *P*-values presented for D2M+ *vs* D2M−.

Number of patients with a missing value: higher education (*n* = 61), no paid work (*n* = 57), current smoking (*n* = 60), symptom duration (*n* = 42), diagnostic delay (*n* = 42), HLA-B27 (*n* = 32), history of EMMs (*n* = 3), history of peripheral manifestations (*n* = 3).

a The presence of ≥1 of the following: high disease activity state, signs suggestive of active disease, or reduced HRQoL.

b Peripheral manifestations included arthritis, enthesitis and dactylitis.

ASAS: Assessment of SpondyloArthritis international Society; D2M: difficult-to-manage; EMM: extra-musculoskeletal manifestation; HRQoL: health-related quality of life.

**Table 3.** Univariable and multivariable analyses of the demographic and disease characteristics associated with the primary ASAS definition of D2M axSpA

Variable	Univariable analysis (*n* = 236)		Multivariable analysis (*n* = 187)	
	OR (95%CI)	*P*	OR (95%CI)	*P*
Female sex	1.9 (0.8–4.7)	0.14	2.2 (0.8–6.1)	0.12
Age	1.0 (1.0–1.0)	0.85	1.0 (1.0–1.0)	0.66
Current smoking	2.4 (0.9–6.3)	0.07	3.1 (1.1–8.7)	0.03
History of EMMs				
Psoriasis	3.0 (1.2–7.6)	0.02	2.8 (1.0–7.6)	0.05
Uveitis	1.9 (0.7–4.8)	0.18	–	–^a^

Multivariable models were always adjusted for age and sex.

Number of patients with a missing value: current smoking (*n* = 48), history of EMMs (*n* = 3).

a Variable not associated with outcome in multivariable models (*P* > 0.05).

ASAS: Assessment of SpondyloArthritis international Society; axSpA: axial spondyloarthritis; D2M: difficult-to-manage; EMM: extra-musculoskeletal manifestation.

Patients who fulfilled the primary ASAS definition showed significantly worse mean scores on several instruments, particularly the ASDAS, BASDAI, back pain VAS, ASAS-HI, SF-36 PCS, PASS-patient and PASS-physician (Table 4). Although these patients scored significantly worse on the PtGA, no statistically significant difference was observed in the mean PhGA between the D2M+ and D2M− groups. Additional observations in the D2M+ groups that were notable, but not statistically significant, were slightly worse outcomes on the SF-36 MCS and a higher percentage of patients with active peripheral manifestations compared with the D2M− groups. Similar results were observed in the analysis of the D2M+ and D2M− groups according to variations A–C.

**Table 4.** Treatment characteristics and outcomes of patients in the D2M and non-D2M groups per definition

Variable	Primary ASAS definition (cumulative^a^) *n* = 236			Variation A (high disease activity) *n* = 263			Variation B (signs of active disease) *n* = 222			Variation C (reduced HRQoL) *n* = 181		
	D2M+ n = 23	D2M− n = 213	*P*	D2M+ n = 23	D2M− n = 240	*P*	D2M+ n = 8	D2M− n = 214	*P*	D2M+ n = 14	D2M− n = 167	*P*
Current medication use												
None, *n* (%)	0 (0.0)	18 (8.5)	0.23	0 (0.0)	19 (7.9)	0.39	0 (0.0)	15 (7.0)	1.00	0 (0.0)	13 (7.8)	0.60
NSAID, *n* (%)	17 (73.9)	140 (65.7)	0.43	17 (73.9)	156 (65.0)	0.39	6 (75.0)	143 (66.8)	1.00	10 (71.4)	107 (64.1)	0.77
csDMARD, *n* (%)	5 (21.7)	22 (10.3)	0.16	5 (21.7)	24 (10.0)	0.15	2 (25.0)	21 (9.8)	0.20	3 (21.4)	19 (11.4)	0.38
bDMARD: TNFi, *n* (%)	13 (56.5)	95 (44.6)	0.28	13 (56.5)	110 (45.8)	0.33	3 (37.5)	101 (47.2)	0.73	7 (50.0)	80 (47.9)	0.88
bDMARD: IL-17i, *n* (%)	5 (21.7)	21 (9.9)	0.15	5 (21.7)	22 (9.2)	0.07	3 (37.5)	22 (10.3)	0.05	3 (21.4)	19 (11.4)	0.38
bDMARD: other, *n* (%)	1 (4.3)	4 (1.9)	0.40	1 (4.3)	4 (1.7)	0.37	0 (0.0)	4 (1.9)	1.00	1 (7.1)	3 (1.8)	0.28
tsDMARD, *n* (%)	4 (17.4)	1 (0.5)	<0.001	4 (17.4)	1 (0.4)	<0.001	2 (25.0)	3 (1.4)	0.01	3 (21.4)	2 (1.2)	<0.01
Systemic glucocorticoid, *n* (%)	1 (4.3)	3 (1.4)	0.34	1 (4.3)	3 (1.3)	0.31	1 (12.5)	2 (0.9)	0.10	1 (7.1)	3 (1.8)	0.28
ASDAS	2.9 (0.5)	2.5 (1.0)	<0.01	2.9 (0.5)	2.4 (1.0)	<0.001	3.3 (0.6)	2.3 (1.0)	<0.01	2.8 (0.4)	2.3 (1.0)	<0.001
BASDAI, 0–10	6.3 (1.3)	4.5 (2.4)	<0.001	6.3 (1.3)	4.3 (2.4)	<0.001	6.9 (1.7)	4.2 (2.4)	<0.01	6.2 (1.1)	4.1 (2.4)	<0.001
CRP, mg/L	2.9 (3.2)	5.5 (8.3)	0.13	2.9 (3.2)	5.1 (7.9)	0.19	5.5 (4.2)	5.5 (8.3)	1.00	1.9 (1.5)	4.8 (6.8)	<0.001
CRP ≥5mg/L, *n* (%)	4 (17.4)	73 (34.3)	0.10	4 (17.4)	73 (30.4)	0.19	4 (50.0)	73 (34.1)	0.45	1 (7.1)	49 (29.3)	0.12
Back pain VAS, 0–10	6.8 (1.3)	5.1 (2.9)	<0.001	6.8 (1.3)	4.8 (2.8)	<0.001	7.1 (1.7)	4.8 (2.8)	0.01	6.9 (1.1)	4.6 (2.8)	<0.001
ASAS-HI, 0–17	8.2 (3.6)	5.5 (3.8)	<0.01	8.2 (3.6)	5.5 (3.8)	<0.01	8.3 (4.1)	5.5 (3.8)	0.06	9.1 (3.3)	5.5 (3.8)	<0.001
SF-36 PCS, 0–100	34.4 (5.9)	41.0 (9.6)	<0.001	34.4 (5.9)	41.2 (9.5)	<0.001	36.0 (6.5)	40.9 (9.6)	0.18	32.2 (4.6)	41.1 (9.5)	<0.001
SF-36 MCS, 0–100	44.0 (13.6)	48.8 (10.5)	0.08	44.0 (13.6)	48.8 (10.4)	0.07	36.9 (11.2)	49.6 (10.1)	<0.01	45.2(14.0)	48.7 (10.6)	0.26
PtGA, 0–10	6.5 (1.4)	4.7 (2.8)	<0.001	6.5 (1.4)	4.5 (2.8)	<0.001	6.6 (1.8)	4.4 (2.8)	0.03	6.8 (1.4)	4.2 (2.8)	<0.001
PASS-patient, *n* (%)	5 (35.7)	88 (71.0)	0.01	5 (35.7)	89 (71.2)	0.01	2 (40.0)	89 (68.5)	0.33	3 (25.0)	91 (71.7)	<0.01
PhGA, 0–10	2.6 (1.8)	2.0 (1.7)	0.13	2.6 (1.8)	1.9 (1.7)	0.08	2.6 (1.6)	2.1 (1.7)	0.38	2.6 (1.5)	2.0 (1.7)	0.19
PASS-physician, *n* (%)	7 (63.6)	101 (91.0)	0.02	7 (63.6)	101 (91.0)	0.02	1 (25.0)	106 (90.6)	<0.01	6 (66.7)	100 (90.9)	0.06
Active peripheral manifestations^b^, *n* (%)	5 (26.3)	26 (15.8)	0.33	5 (26.3)	26 (14.3)	0.18	5 (83.3)	26 (13.8)	<0.001	4 (30.8)	20 (14.2)	0.12
BSA ≥3%, *n* (%)	0 (0.0)	1 (0.7)	1.00	0 (0.0)	1 (0.6)	1.00	0 (0.0)	1 (0.6)	1.00	0 (0.0)	1 (0.7)	1.00

All values presented as mean (s.d.), unless otherwise indicated. *P*-values presented for D2M+ *vs* D2M−.

Number of patients with a missing value: ASAS-HI (*n* = 92), SF-36 (*n* = 77), PASS-patient (*n* = 124), PhGA (*n* = 47), PASS-physician (*n* = 141), active peripheral manifestations (*n* = 62), BSA (*n* = 76).

a The presence of ≥1 of the following: high disease activity state, signs suggestive of active disease, or reduced HRQoL.

b Peripheral manifestations included arthritis, enthesitis and dactylitis.

ASAS: Assessment of SpondyloArthritis international Society; ASAS-HI: ASAS Health Index; ASDAS: Axial Spondyloarthritis Disease Activity Score; bDMARD: biological DMARD; BSA: psoriasis body surface area; csDMARD: conventional synthetic DMARD; D2M: difficult-to-manage; HRQoL: health-related quality of life; IL-17i: IL-17 inhibitor; PASS: patient/physician acceptable symptom state; PhGA: physician global assessment; PtGA: patient global assessment; SF-36 MCS: 36-Item Short Form Health Survey Mental Component Summary; SF-36 PCS: SF-36 Physical Component Summary; TNFi: TNF inhibitor; tsDMARD: targeted synthetic DMARD; VAS: visual analogue scale.

Characteristics associated with TR axSpA were not explored due to the small number of patients fulfilling this definition (*n* = 4).

### Sensitivity analyses

The completer analysis resulted in a similar prevalence of D2M axSpA across all definitions, and comparable trends in the demographic, disease and treatment characteristics of patients classified as D2M+ and D2M− (Supplementary Figs S1 and S2 and Supplementary Tables S2 and S3, available at *Rheumatology* online). In the multivariable analysis of completers, having no paid work, a history of psoriasis and a history of uveitis were factors found to be associated with having D2M axSpA, while current smoking was not (Supplementary Table S4, available at *Rheumatology* online).

Lowering the cut-off of the PtGA/PhGA had minimal impact on the prevalence of D2M and TR axSpA, despite a larger proportion of patients fulfilling the ‘problematic management’ domain (Supplementary Table S5, available at *Rheumatology* online). When the PASS was used instead of the PtGA/PhGA, a slightly lower prevalence of D2M axSpA was observed. For example, only 10 of 130 (7.7%) patients with data available on the PASS were considered to have D2M axSpA according to the primary ASAS definition, in comparison to 9.7% in the main analysis (Supplementary Table S6, available at *Rheumatology* online). None of the patients were classified as having TR axSpA by the PASS-based definitions. On further exploration, four additional patients in this population would have fulfilled the primary ASAS definition if the PhGA/PtGA had been used instead of the PASS (*n* = 14/130, 10.8%) (Supplementary Fig. S3, available at *Rheumatology* online). Patients fulfilling this PtGA/PhGA-based definition, but not the PASS-based definition, had similar outcomes and disease burden as those fulfilling both definitions (Supplementary Table S7, available at *Rheumatology* online).

## Discussion

In this study, we applied the recently established ASAS definition of D2M axSpA in a real-world clinical setting, offering insight into the implications of applying this definition in clinical practice. In our multicentre SpA registry, 10% of patients had D2M axSpA according to the ASAS definition, and 2% were considered to have TR disease.

Amongst the domains of D2M axSpA, we observed that ‘treatment failure’ was the most challenging to fulfil. In the ‘insufficient control of signs and symptoms’ domain, most patients qualified based on instruments that are (partially) patient-reported, namely the ASDAS, ASAS-HI or SF-36, rather than objective signs. For the ‘problematic management’ domain, lowering the PtGA/PhGA cut-off had minimal impact on the resulting prevalence of D2M axSpA due to many patients already fulfilling the other domains. With respect to selecting an instrument to measure this domain, PtGA/PhGA seemed more sensitive than PASS to determine D2M axSpA.

Three studies on the epidemiology of D2M axSpA have recently been published [27–29]. Notably, two of these, both conducted in France, used exclusively the ‘treatment failure’ domain in their definitions [27, 28]. One study defined ‘treatment failure’ less stringently (failure of ≥3 b/tsDMARDs of any MoA), and found a higher percentage of patients fulfilling this domain, with 2115 cases in a cohort of 10 798 (19.6%) patients, in contrast to 9.9–11.6% found in our cohort [27]. The other French study applied the same criterion as ours (failure of ≥2 b/tsDMARDs of different MoA), but also reported a higher percentage of ‘treatment failure’ with 88 cases in a cohort of 311 (28.3%) patients [28]. A third Turkish study, which considered all three domains of D2M axSpA, found a prevalence of 38 cases in a cohort of 166 (22.9%) patients, compared with 9.7% found in our cohort based on all domains [29]. Again, however, this study applied less stringent subcriteria (e.g. an ASDAS cut-off of ≥1.3 instead of ≥2.1 to define insufficient disease control), alongside indicators that are not endorsed by ASAS (e.g. regular or frequent NSAID use), which may explain the observed discrepancies in prevalence [29]. Additionally, potential regional differences in treatment intensification protocols might have led to differences in the prevalence of D2M axSpA.

We observed considerable similarities with other studies regarding the patient characteristics associated with D2M axSpA, particularly current smoking and a history of psoriasis [27–29]. While other characteristics, including female sex, a history of EMMs other than psoriasis or a history of peripheral manifestations, have also been associated with (components of) D2M axSpA previously, these were not found to be significant in our multivariable analysis [27–31]. Nevertheless, considering that relevant effect sizes were still observed and that the completer analysis showed slightly different results (i.e. association of having no paid work, and a history of psoriasis or uveitis with D2M axSpA), these characteristics should not yet be excluded. Our findings are also consistent with previous studies outlining the relation between smoking and higher disease activity in patients with axSpA, the association of psoriasis and peripheral manifestations with more frequent bDMARD use, and the tendency of female patients to experience more pronounced limitations in physical function [32–37]. Smoking, being a modifiable risk factor, might be of particular interest in the clinical setting. Furthermore, while the relation between having no paid work and D2M axSpA specifically has not been described before, its potential association is consistent with previous findings of higher disease burden amongst unemployed patients [38]. Importantly, however, not having paid work may also be a consequence of difficulties surrounding disease management, rather than an independent predictive factor of one’s disease course. Additional associated characteristics previously reported include obesity, hypertension, depression and pain sensitization syndromes [27–29]. These characteristics have a plausible connection with problematic disease management and may influence patient-reported outcomes (PROs), but could not be explored in our analysis as the relevant data were unavailable.

Our results also emphasize that patients affected by D2M axSpA may experience higher disease burden compared with the broader axSpA patient population, as highlighted previously [29]. Poorer outcomes on diverse PROs (including outcomes not included as indicators of D2M axSpA in this study) were found, which corresponds with observations of impaired function and participation made in patients with D2T RA [14]. Although the exact nature of these limitations cannot be derived from our results, these findings nevertheless reinforce the importance of early identification and adequate management of patients with D2M axSpA.

A prominent strength of this study was our application of the ASAS definition, making this one of the first studies to explore all domains included in the D2M axSpA concept in clinical practice. This is an important contrast with previous studies which applied non-endorsed definitions. Furthermore, we included several variations of the ASAS definition in our analyses, providing comprehensive insight into specific components of this definition that contribute to the classification of D2M axSpA. Finally, our data originated from a comprehensive daily practice registry including data from both university and general hospitals, and patients from diverse backgrounds.

This study also has several limitations. Firstly, its cross-sectional design allows for the inference of associations between variables, but not causality. Secondly, certain observations made were based on a limited sample size. Despite this, our sensitivity analyses showed comparable results to the main analysis. Thirdly, proxy instruments (PtGA/PhGA) were employed to define the ‘problematic management’ domain, as specific indicators were not available in SpA-Net. While these are widely used instruments in axSpA that reflect the disease management process, the arbitrary nature of the cut-off value applied may have introduced a degree of uncertainty into the ‘problematic management’ domain, considering that the number of patients classified as having D2M axSpA is inherently dependent on the chosen cut-off value [39, 40]. Other potential indicators of a problematic disease course, such as use of analgesics, were unavailable in SpA-Net. Finally, several factors may have skewed the prevalence of D2M axSpA. For example, the PtGA, used for the ‘problematic management’ domain, overlaps with one of the subcriteria included in the ‘insufficient control of signs and symptoms’ domain (i.e. ASDAS, of which the PtGA is a component). Patients with an elevated ASDAS were, by definition, also more likely to have an elevated PtGA, thereby potentially leading to an overestimation of patients classified as having D2M axSpA. An overestimation of the prevalence of TR axSpA specifically may also have resulted from our inability to exclude alternative reasons for non-response that are highlighted in the ASAS definition, such as concomitant conditions or non-compliance, as data on these aspects were not available in SpA-Net [16]. Conversely, the absence of information on certain indicators of disease control in SpA-Net stipulated in the ASAS definition, such as on the presence of active IBD or uveitis, MRI to ascertain active inflammation in the sacroiliac joints and/or spine, or radiographs to assess rapid progression in structural damage of the spine, may have led to an extent of underestimation as well [16].

Overall, this exploratory study contributes to improved awareness of the prevalence of D2M axSpA and potentially associated patient characteristics in clinical practice, and serves as a stepping stone towards further research on this topic. Specifically, research with larger sample sizes and more comprehensive, prospective data (including data on EMMs and imaging) collected for the specific purpose of investigating D2M axSpA is necessary to confirm our results, alongside the development of optimized management strategies for patients with D2M axSpA.

In conclusion, D2M axSpA affects approximately one in 10 patients with axSpA according to the ASAS definition. PROs contribute importantly to the classification of D2M axSpA, whereas treatment failure is the most prominent limiting factor. Current smoking and a history of psoriasis are associated characteristics with having D2M axSpA.

## Supplementary Material

keaf120_Supplementary_Data

## Data Availability

The data that supports the findings of this study are available from the corresponding author upon reasonable request.
