# Supplementary material for: Exploring difficult-to-manage axial spondyloarthritis: results from a Dutch clinical practice registry
Source: Rheumatology (Oxford). 2025 Feb 28;64(6):3816–25. doi: 10.1093/rheumatology/keaf120 (PMC12107025; doi:10.1093/rheumatology/keaf120)
Supplement: keaf120_Supplementary_Data [file keaf120_supplementary_data.docx]

Supplementary material


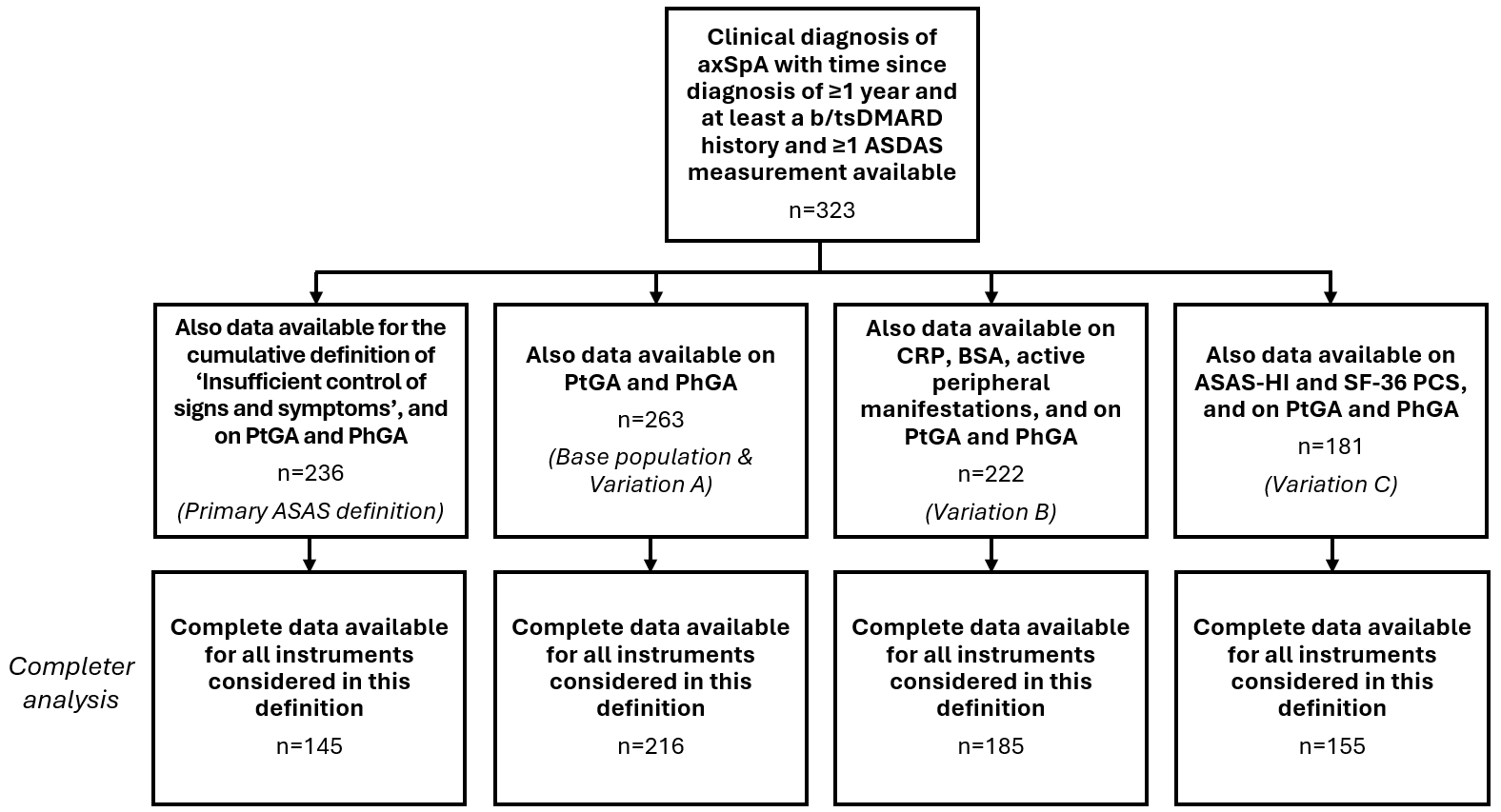


***Supplementary Figure S1.*** Flowchart of the inclusion of patient data from SpA-Net.

The analysis of each definition included patients with available data on any of the instruments considered in each domain. Peripheral manifestations included arthritis, enthesitis and dactylitis.

ASAS: Assessment of SpondyloArthritis international Society; ASAS-HI: ASAS-Health Index; ASDAS: Axial Spondyloarthritis Disease Activity Score; axSpA: axial spondyloarthritis; BSA: psoriasis body surface area; CRP: C-reactive protein; PhGA: physician global assessment; PtGA: patient global assessment; SF-36 PCS: 36-Item Short Form Health Survey Physical Component Summary.

***Supplementary Table S1.*** Characteristics of patients included in the analysis of each definition

| **Variable** | **Population analysed in the primary ASAS definition**  **n=236** | **Population analysed in variation A (base population)**  **n=263** | **Population analysed in variation B**  **n=222** | **Population analysed in variation C**  **n=181** |
| --- | --- | --- | --- | --- |
| Female sex, n (%) | 109 (46.2) | 120 (45.6) | 99 (44.6) | 85 (47.0) |
| Age, years | 52.1 (13.8) | 52.1 (13.8) | 52.2 (13.8) | 51.7 (13.3) |
| Higher education, n (%) | 69 (36.9) | 73 (36.1) | 68 (37.4) | 66 (37.9) |
| No paid work, n (%) | 87 (45.5) | 91 (44.2) | 86 (46.2) | 78 (43.8) |
| Current smoking, n (%) | 42 (22.3) | 44 (21.7) | 38 (20.8) | 34 (19.3) |
| Symptom duration, years | 21.5 (12.7) | 21.7 (12.7) | 21.8 (12.6) | 21.1 (12.6) |
| Diagnostic delay, years | 6.9 (8.8) | 7.0 (9.0) | 7.1 (9.2) | 6.4 (8.4) |
| HLA-B27 positive, n (%) | 151 (72.2) | 166 (71.9) | 136 (69.7) | 113 (69.8) |
| History of EMMs  Psoriasis, n (%)  IBD, n (%)  Uveitis, n (%) | 42 (18.0)  29 (12.4)  57 (24.5) | 44 (16.9)  30 (11.5)  60 (23.1) | 41 (18.6)  26 (11.8)  48 (21.8) | 37 (20.6)  23 (12.8)  43 (23.9) |
| History of peripheral manifestations^†^, n (%) | 105 (45.1) | 113 (43.5) | 104 (47.3) | 94 (52.2) |
| Current medication use  None, n (%)  NSAID, n (%)  csDMARD, n (%)  bDMARD: TNFi, n (%)  bDMARD: IL-17i, n (%)  bDMARD: other, n (%)  tsDMARD, n (%)  Systemic glucocorticoid,  n (%) | 18 (7.6)  157 (66.5)  27 (11.4)  108 (45.8)  26 (11.0)  5 (2.1)  5 (2.1)  4 (1.7) | 19 (7.2)  173 (65.8)  29 (11.0)  123 (46.8)  27 (10.3)  5 (1.9)  5 (1.9)  4 (1.5) | 15 (6.8)  149 (67.1)  23 (10.4)  104 (46.8)  25 (11.3)  4 (1.8)  5 (2.3)  3 (1.4) | 13 (7.2)  117 (64.6)  22 (12.2)  87 (48.1)  22 (12.2)  4 (2.2)  5 (2.8)  4 (2.2) |
| ASDAS | 2.5 (1.0) | 2.4 (1.0) | 2.4 (1.0) | 2.3 (1.0) |
| BASDAI, 0-10 | 4.7 (2.4) | 4.5 (2.4) | 4.3 (2.4) | 4.3 (2.4) |
| CRP, mg/L | 5.3 (8.0) | 4.9 (7.6) | 5.5 (8.2) | 4.6 (6.6) |
| CRP ≥5mg/L, n (%) | 77 (32.6) | 77 (29.3) | 77 (34.7) | 50 (27.6) |
| PtGA, 0-10 | 4.9 (2.8) | 4.7 (2.8) | 4.5 (2.8) | 4.4 (2.8) |
| Back pain VAS, 0-10 | 5.2 (2.8) | 5.0 (2.8) | 4.9 (2.8) | 4.8 (2.7) |
| ASAS-HI, 0-17 | 5.8 (3.8) | 5.8 (3.9) | 5.7 (3.8) | 5.8 (3.9) |
| SF-36 PCS, 0-100 | 40.3 (9.4) | 40.6 (9.4) | 40.7 (9.5) | 40.4 (9.5) |
| SF-36 MCS, 0-100 | 48.3 (10.9) | 48.4 (10.8) | 49.1 (10.4) | 48.4 (10.9) |
| PASS-patient, n (%) | 93 (67.4) | 94 (67.6) | 91 (67.4) | 94 (67.6) |
| PhGA, 0-10 | 2.1 (1.7) | 2.0 (1.7) | 2.1 (1.7) | 2.0 (1.7) |
| PASS-physician, n (%) | 108 (88.5) | 108 (88.5) | 107 (88.4) | 106 (89.1) |
| Active peripheral manifestations^†^, n (%) | 31 (16.8) | 31 (15.4) | 31 (15.9) | 24 (15.6) |
| BSA ≥3%, n (%) | 1 (0.6) | 1 (0.5) | 1 (0.5) | 1 (0.7) |
| All values presented as mean (SD), unless otherwise indicated.  ^†^ Peripheral manifestations included arthritis, enthesitis and dactylitis.  Number of patients with a missing value: Higher education (n=61), No paid work (n=57), Current smoking (n=60), Symptom duration (n=42), Diagnostic delay (n=42), HLA-B27 (n=32), History of EMMs (n=3), History of peripheral manifestations (n=3), ASAS-HI (n=92), SF-36 (n=77), PASS-patient (n=124), PhGA (n=47), PASS-physician (n=141), Active peripheral manifestations (n=62), BSA (n=76).  ASAS: Assessment of SpondyloArthritis international Society; ASAS-HI: ASAS-Health Index; ASDAS: Axial Spondyloarthritis Disease Activity Score; BASDAI: Bath Ankylosing Spondylitis Disease Activity Index; bDMARD: biological disease-modifying anti-rheumatic drug; BSA: psoriasis body surface area; CRP: C-reactive protein; csDMARD: conventional synthetic DMARD; D2M: difficult-to-manage; EMM: extra-musculoskeletal manifestation; HLA-B27: human leukocyte antigen B27; IBD: inflammatory bowel disease; IL-17i: interleukin-17 inhibitor; NSAID: non-steroidal anti-inflammatory drug; PASS: patient/physician acceptable symptom state; PhGA: physician global assessment; PtGA: patient global assessment; SF-36 MCS: 36-Item Short Form Health Survey Mental Component Summary; SF-36 PCS: SF-36 Physical Component Summary; TNFi: tumour necrosis factor inhibitor; tsDMARD: targeted synthetic DMARD; VAS: visual analogue scale. | | | | |

**
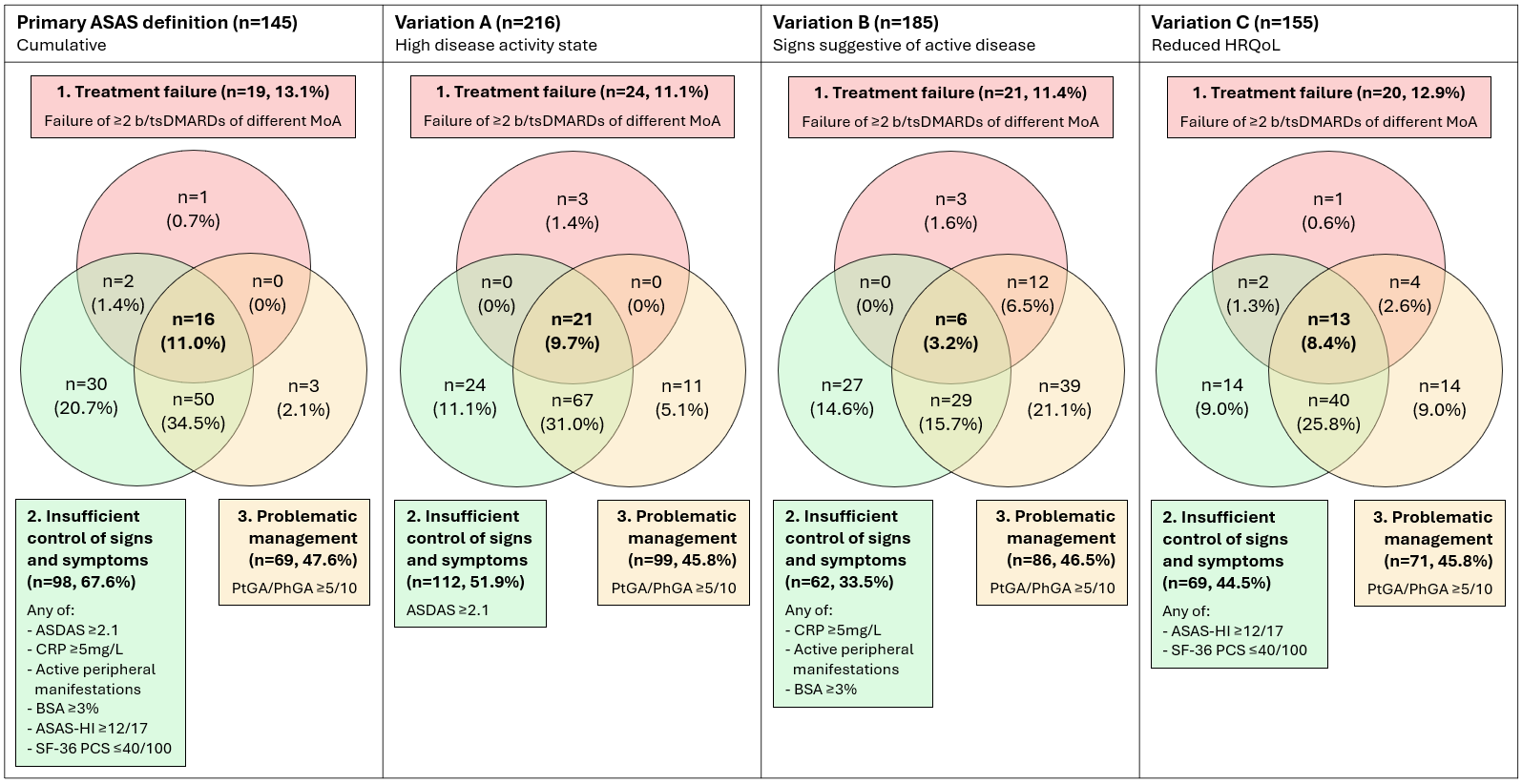
**

***Supplementary Figure S2.*** Sensitivity analysis: Completer analysis of the percentages of patients fulfilling the primary ASAS definition and three explored variations, as well as the individual domains within the definitions.

Percentages of patients fulfilling each individual domain per definition are presented in the respective boxes. For example, 19 of 145 (13.1%) patients fulfilled the ‘treatment failure’ domain in total in the population considered in the completer analysis of the primary ASAS definition, irrespective of the other two domains. The completer analysis of each definition included patients with complete data available for every instrument considered in each domain. Peripheral manifestations included arthritis, enthesitis and dactylitis.

ASAS: Assessment of SpondyloArthritis international Society; ASAS-HI: ASAS-Health Index; ASDAS: Axial Spondyloarthritis Disease Activity Score; bDMARD: biological disease-modifying anti-rheumatic drug; BSA: psoriasis body surface area; CRP: C-reactive protein; HRQoL: health-related quality of life; MoA: mode of action; PhGA: physician global assessment; PtGA: patient global assessment; SF-36 PCS: 36-Item Short Form Health Survey Physical Component Summary; tsDMARD: targeted synthetic DMARD.

***Supplementary Table S2.*** Sensitivity analysis: Completer analysis of the demographic and disease characteristics of patients in the D2M and non-D2M groups per definition

| **Variable** | **Primary ASAS definition**  **(cumulative^†^)**  **n=145** | | | **Variation A**  **(high disease activity)**  **n=216** | | | **Variation B**  **(signs of active disease)**  **n=185** | | | **Variation C**  **(reduced HRQoL)**  **n=155** | | | |
| --- | --- | --- | --- | --- | --- | --- | --- | --- | --- | --- | --- | --- | --- |
|  | **D2M+ n=16** | **D2M- n=129** | ***p*** | **D2M+ n=21** | **D2M- n=195** | ***p*** | **D2M+**  **n=6** | **D2M- n=179** | ***p*** | **D2M+ n=13** | **D2M- n=142** | ***p*** |  |
| Female sex, n (%) | 10  (62.5) | 54  (41.9) | 0.12 | 12  (57.1) | 83  (42.6) | 0.20 | 4  (66.7) | 77  (43.0) | 0.41 | 9  (69.2) | 58  (40.8) | 0.05 |  |
| Age, years | 54.2  (13.0) | 52.0  (13.5) | 0.54 | 52.1  (13.3) | 52.1  (13.7) | 0.99 | 57.9  (9.9) | 52.4  (13.5) | 0.32 | 52.8  (14.0) | 51.9  (13.2) | 0.82 |  |
| Higher education,  n (%) | 7  (43.8) | 51  (39.5) | 0.75 | 7  (36.8) | 62  (37.3) | 0.97 | 2  (33.3) | 62  (38.8) | 1.00 | 6  (46.2) | 55  (38.7) | 0.60 |  |
| No paid work, n (%) | 10  (62.5) | 53  (41.1) | 0.10 | 12  (63.2) | 70  (42.2) | 0.08 | 4  (66.7) | 71  (44.4) | 0.41 | 9  (69.2) | 57  (40.1) | 0.04 |  |
| Current smoking,  n (%) | 5  (31.3) | 21  (16.3) | 0.17 | 8  (42.1) | 31  (18.9) | 0.03 | 3  (50.0) | 27  (17.0) | 0.07 | 4  (30.8) | 26  (18.4) | 0.28 |  |
| Symptom duration, years | 24.7  (16.4) | 21.3  (12.5) | 0.34 | 23.1  (15.2) | 21.6  (12.3) | 0.62 | 18.4  (12.7) | 22.1  (12.9) | 0.53 | 24.4  (17.7) | 21.4  (12.3) | 0.58 |  |
| Diagnostic delay, years | 9.5  (14.4) | 5.8  (7.7) | 0.34 | 9.0  (13.3) | 6.7  (8.7) | 0.46 | 3.9  (3.1) | 7.0  (9.2) | 0.45 | 10.9  (15.8) | 5.9  (7.7) | 0.30 |  |
| HLA-B27 positive, n (%) | 9  (60.0) | 78  (67.8) | 0.57 | 12  (60.0) | 117  (68.4) | 0.45 | 3  (50.0) | 103  (65.6) | 0.42 | 7  (58.3) | 87  (68.5) | 0.52 |  |
| History of EMMs  Psoriasis, n (%)    IBD, n (%)    Uveitis, n (%) | 8  (50.0)  1  (6.3)  7  (43.8) | 26  (20.2)  15  (11.6)  25  (19.4) | 0.01  1.00  0.05 | 8  (38.1)  1  (4.8)  7  (33.3) | 32  (16.4)  20  (10.3)  41  (21.0) | 0.03  0.70  0.27 | 3  (50.0)  1  (16.7)  3  (50.0) | 35  (19.6)  20  (11.2)  36  (20.1) | 0.10  0.52  0.11 | 7  (53.8)  1  (7.7)  5  (38.5) | 27  (19.0)  15  (10.6)  30  (21.1) | 0.01  1.00  0.17 |  |
| History of peripheral manifestations^‡^,  n (%) | 11  (68.8) | 65  (50.4) | 0.17 | 12  (57.1) | 90  (46.2) | 0.34 | 5  (83.3) | 89  (49.7) | 0.21 | 10  (76.9) | 70  (49.3) | 0.06 |  |
| All values presented as mean (SD) unless otherwise indicated. P-values presented for D2M+ vs. D2M-.  ^†^ Presence of ≥1 of the following: high disease activity, signs suggestive of active disease, or reduced HRQoL.  ^‡^ Peripheral manifestations included arthritis, enthesitis and dactylitis.  Number of patients with a missing value: Higher education (n=31), No paid work (n=31), Current smoking (n=33), Symptom duration (n=24), Diagnostic delay (n=24), HLA-B27 (n=25).  ASAS: Assessment of SpondyloArthritis international Society; D2M: difficult-to-manage; EMM: extra-musculoskeletal manifestation; HLA-B27: human leukocyte antigen B27; HRQoL: health-related quality of life; IBD: inflammatory bowel disease. | | | | | | | | | | | | | |

***Supplementary Table S3.*** Sensitivity analysis: Completer analysis of the treatment characteristics and outcomes of patients in the D2M and non-D2M groups per definition

| **Variable** | **Primary ASAS definition**  **(cumulative^†^)**  **n=145** | | | **Variation A**  **(high disease activity)**  **n=216** | | | **Variation B**  **(signs of active disease)**  **n=185** | | | **Variation C**  **(reduced HRQoL)**  **n=155** | | | |
| --- | --- | --- | --- | --- | --- | --- | --- | --- | --- | --- | --- | --- | --- |
|  | **D2M+ n=16** | **D2M- n=129** | ***p*** | **D2M+ n=21** | **D2M- n=195** | ***p*** | **D2M+**  **n=6** | **D2M- n=179** | ***p*** | **D2M+ n=13** | **D2M- n=142** | ***p*** |  |
| Current medication use  None, n (%)    NSAID, n (%)  csDMARD, n (%)  bDMARD: TNFi,  n (%)  bDMARD: IL-17i,  n (%)  bDMARD: other,  n (%)  tsDMARD, n (%)  Systemic  glucocorticoid, n (%) | 0  (0.0)  13  (81.3)  4  (25.0)  7  (43.8)  4  (25.0)  1  (6.3)  4  (25.0)  1  (6.3) | 9  (7.0)  87  (67.4)  11  (8.5)  62  (48.1)  17  (13.2)  2  (1.6)  1  (0.8)  2  (1.6) | 0.60  0.39  0.06  0.74  0.25  0.30  <0.001  0.30 | 0  (0.0)  17  (81.0)  4  (19.0)  11  (52.4)  5  (23.8)  1  (4.8)  4  (19.0)  1  (4.8) | 13  (6.7)  128  (65.6)  19  (9.7)  100  (51.3)  20  (10.3)  3  (1.5)  1  (0.5)  2  (1.0) | 0.62  0.16  0.25  0.92  0.08  0.34  <0.001  0.27 | 0  (0.0)  5  (83.3)  2  (33.3)  1  (16.7)  3  (50.0)  0  (0.0)  2  (33.3)  1  (16.7) | 11  (6.1)  125  (69.8)  18  (10.1)  89  (49.7)  21  (11.7)  3  (1.7)  3  (1.7)  2  (1.1) | 1.00  0.67  0.13  0.21  0.03  1.00  0.01  0.09 | 0  (0.0)  10  (76.9)  3  (23.1)  6  (46.2)  3  (23.1)  1  (7.7)  3  (23.1)  1  (7.7) | 9  (6.3)  94  (66.2)  14  (9.9)  70  (49.3)  18  (12.7)  3  (2.1)  2  (1.4)  2  (1.4) | 1.00  0.55  0.16  0.83  0.39  0.30  <0.01  0.23 |  |
| ASDAS | 2.8  (0.4) | 2.1  (1.0) | <0.001 | 2.9  (0.5) | 2.2  (1.0) | <0.001 | 3.1  (0.3) | 2.1  (0.9) | <0.001 | 2.8  (0.4) | 2.1  (0.9) | <0.001 |  |
| BASDAI, 0-10 | 6.1  (1.2) | 3.8  (2.3) | <0.001 | 6.3  (1.4) | 3.9  (2.3) | <0.001 | 6.6  (1.4) | 3.9  (2.2) | <0.01 | 6.2  (1.1) | 3.8  (2.3) | <0.001 |  |
| CRP, mg/L | 2.4  (2.1) | 4.2  (6.0) | 0.26 | 3.0  (3.3) | 4.8  (7.7) | 0.29 | 3.8  (2.6) | 3.8  (5.3) | 0.99 | 1.9  (1.6) | 4.3  (5.9) | 0.15 |  |
| CRP ≥5mg/L, n (%) | 2  (12.5) | 31  (24.0) | 0.53 | 4  (19.0) | 52  (26.7) | 0.45 | 2  (33.3) | 40  (22.3) | 0.62 | 1  (7.7) | 37  (26.1) | 0.19 |  |
| Back pain VAS, 0-10 | 6.8  (1.1) | 4.3  (2.7) | <0.001 | 6.9  (1.3) | 4.4  (2.8) | <0.001 | 6.8  (1.5) | 4.4  (2.7) | 0.01 | 7.0  (1.0) | 4.3  (2.7) | <0.001 |  |
| ASAS-HI, 0-17 | 8.3  (3.1) | 5.1  (3.7) | <0.01 | 7.8  (3.4) | 5.1  (3.7) | <0.01 | 9.6  (2.7) | 5.3  (3.7) | 0.01 | 8.7  (3.1) | 5.1  (3.6) | <0.001 |  |
| **Variable** | **Primary ASAS definition**  **(cumulative^†^)**  **n=145** | | | **Variation A**  **(high disease activity)**  **n=216** | | | **Variation B**  **(signs of active disease)**  **n=185** | | | **Variation C**  **(reduced HRQoL)**  **n=155** | | |  |
|  | **D2M+ n=16** | **D2M- n=129** | ***p*** | **D2M+ n=21** | **D2M- n=195** | ***p*** | **D2M+**  **n=6** | **D2M- n=179** | ***p*** | **D2M+ n=13** | **D2M- n=142** | ***p*** |  |
| SF-36 PCS, 0-100 | 34.4  (5.6) | 42.7  (9.2) | <0.001 | 34.9  (5.8) | 42.2  (9.4) | <0.01 | 34.9  (6.3) | 41.8  (9.4) | 0.08 | 32.6  (4.5) | 42.7  (9.1) | <0.001 |  |
| SF-36 MCS, 0-100 | 45.6  (12.9) | 49.7  (9.9) | 0.13 | 45.5  (12.5) | 49.7  (10.1) | 0.11 | 36.0  (12.0) | 49.7  (9.9) | <0.01 | 47.2  (12.5) | 49.7  (10.3) | 0.42 |  |
| PtGA, 0-10 | 6.4  (1.4) | 3.9  (2.8) | <0.001 | 6.5  (1.4) | 4.0  (2.8) | <0.001 | 6.5  (1.6) | 4.1  (2.7) | 0.03 | 6.7  (1.4) | 3.8  (2.8) | <0.001 |  |
| PASS-patient, n (%) | 5  (38.5) | 81  (71.7) | 0.02 | 5  (38.5) | 85  (72.0) | 0.02 | 2  (40.0) | 84  (69.4) | 0.33 | 3  (27.3) | 86  (72.9) | <0.01 |  |
| PhGA, 0-10 | 2.6  (1.6) | 1.9  (1.6) | 0.10 | 2.6  (1.8) | 1.9  (1.7) | 0.08 | 3.2  (1.5) | 2.1  (1.7) | 0.11 | 2.6  (1.5) | 1.9  (1.6) | 0.13 |  |
| PASS-physician,  n (%) | 7  (63.6) | 98  (91.6) | 0.02 | 7  (63.6) | 101  (91.0) | 0.02 | 1  (25.0) | 106  (90.6) | <0.01 | 6  (66.7) | 100  (90.9) | 0.06 |  |
| Active peripheral manifestations^‡^,  n (%) | 5  (31.3) | 14  (10.9) | 0.04 | 5  (26.3) | 22  (12.4) | 0.15 | 5  (83.3) | 21  (11.7) | <0.001 | 4  (30.8) | 16  (12.0) | 0.08 |  |
| BSA ≥3%, n (%) | 0  (0.0) | 1  (0.8) | 1.00 | 0  (0.0) | 1  (0.6) | 1.00 | 0  (0.0) | 1  (0.6) | 1.00 | 0  (0.0) | 1  (0.8) | 1.00 |  |
| All values presented as mean (SD), unless otherwise indicated. P-values presented for D2M+ vs. D2M-.  ^†^ Presence of ≥1 of the following: high disease activity, signs suggestive of active disease, or reduced HRQoL.  ^‡^ Peripheral manifestations included arthritis, enthesitis and dactylitis.  Number of patients with a missing value: ASAS-HI (n=61), SF-36 (n=49), PASS-patient (n=85), PASS-physician (n=94), Active peripheral symptoms (n=19), BSA (n=30).  ASAS: Assessment of SpondyloArthritis international Society; ASAS-HI: ASAS-Health Index; ASDAS: Axial Spondyloarthritis Disease Activity Score; BASDAI: Bath Ankylosing Spondylitis Disease Activity Index; bDMARD: biological disease-modifying anti-rheumatic drug; BSA: psoriasis body surface area; CRP: C-reactive protein; csDMARD: conventional synthetic DMARD; D2M: difficult-to-manage; HRQoL: health-related quality of life; IL-17i: interleukin-17 inhibitor; NSAID: non-steroidal anti-inflammatory drug; PASS: patient/physician acceptable symptom state; PhGA: physician global assessment; PtGA: patient global assessment; SF-36 MCS: 36-Item Short Form Health Survey Mental Component Summary; SF-36 PCS: SF-36 Physical Component Summary; TNFi: tumour necrosis factor inhibitor; tsDMARD: targeted synthetic DMARD; VAS: visual analogue scale. | | | | | | | | | | | | |  |

***Supplementary Table S4.*** Sensitivity analysis: Univariable and multivariable analyses of the demographic and disease characteristics associated with the primary ASAS definition of D2M axSpA, completer analysis

| **Variable** | **Univariable analysis (n=145)** | | **Multivariable analysis (n=145)** | |
| --- | --- | --- | --- | --- |
|  | **OR (95%CI)** | ***p*** | **OR (95%CI)** | ***p*** |
| Female sex | 2.3 (0.8-6.8) | 0.12 | 2.6 (0.8-8.1) | 0.11 |
| Age | 1.0 (1.0-1.1) | 0.54 | 1.0 (0.9-1.0) | 0.38 |
| No paid work | 2.4 (0.8-7.0) | 0.11 | 4.3 (1.1-16.7) | 0.04 |
| Current smoking | 2.3 (0.7-7.4) | 0.15 | - ^‡^ | - |
| History of EMMs  Psoriasis  Uveitis | 4.0 (1.4-11.6)  3.2 (1.1-9.5) | 0.01  0.03 | 5.2 (1.5-17.6)  4.7 (1.4-15.8) | 0.01  0.01 |
| History of peripheral manifestations^†^ | 2.2 (0.7-6.6) | 0.17 | - ^‡^ | - |
| Multivariable models were always adjusted for age and sex.  ^†^ Peripheral manifestations included arthritis, enthesitis and dactylitis.  ^‡^ Variable not associated with outcome in multivariable models (p>0.05).  ASAS: Assessment of SpondyloArthritis international Society; axSpA: axial spondyloarthritis; D2M: difficult-to-manage; EMM: extra-musculoskeletal manifestation. | | | | |

***Supplementary Table S5.*** Sensitivity analysis: Percentages of patients fulfilling each explored definition and the individual domains within the definitions, with PtGA/PhGA cut-off of ≥4

| **Domains** | **Definition** |  |  |  |  | |
| --- | --- | --- | --- | --- | --- | --- |
|  | **Primary ASAS definition**  **(Cumulative^†^)**  **n=236** | **Variation A**  **(High disease activity)**  **n=263** | **Variation B**  **(Signs of active disease)**  **n=222** | **Variation C**  **(Reduced HRQoL)**  **n=181** | **Treatment-refractory**  **n=236** | |
| (1) Treatment failure | 26 (11.0) | 26 (9.9) | 23 (10.4) | 21 (11.6) | NA | |
| (2) Insufficient disease control | 188 (79.7) | 150 (57.0) | 99 (44.6) | 93 (51.4) | NA | |
| (3) Problematic management | 161 (68.2) | 172 (65.4) | 139 (62.6) | 109 (60.2) | NA | |
| All domains | 24 (10.2) | 23 (8.7) | 8 (3.6) | 15 (8.3) | 4 (1.7) | |
| All values presented as n (%).  ^†^ Presence of ≥1 of the following: high disease activity, signs suggestive of active disease, or reduced HRQoL.  ASAS: Assessment of SpondyloArthritis international Society; HRQoL: health-related quality of life; NA: not applicable; PhGA: physician global assessment; PtGA: patient global assessment. | | | | | |  |

***Supplementary Table S6.*** Sensitivity analysis: Percentages of patients fulfilling each explored definition and the individual domains within the definitions, using the PASS-patient/physician for the ‘problematic management’ domain

| **Domains** | **Definition** |  |  |  |  | |
| --- | --- | --- | --- | --- | --- | --- |
|  | **Primary ASAS definition**  **(Cumulative^†^)**  **n=130** | **Variation A**  **(High disease activity)**  **n=130** | **Variation B**  **(Signs of active disease)**  **n=128** | **Variation C**  **(Reduced HRQoL)**  **n=129** | **Treatment-refractory**  **n=130** | |
| (1) Treatment failure | 17 (13.1) | 17 (13.1) | 16 (12.5) | 17 (13.2) | NA | |
| (2) Insufficient disease control | 87 (66.9) | 67 (51.5) | 45 (35.2) | 61 (47.3) | NA | |
| (3) Problematic management | 49 (37.7) | 49 (37.7) | 48 (37.5) | 48 (37.2) | NA | |
| All domains | 10 (7.7) | 10 (7.7) | 4 (3.1) | 9 (7.0) | 0 (0.0) | |
| All values presented as n (%).  ^†^ Presence of ≥1 of the following: high disease activity, signs suggestive of active disease, or reduced HRQoL.  ASAS: Assessment of SpondyloArthritis international Society; HRQoL: health-related quality of life; NA: not applicable; PASS: patient/physician acceptable symptom state. | | | | | |  |


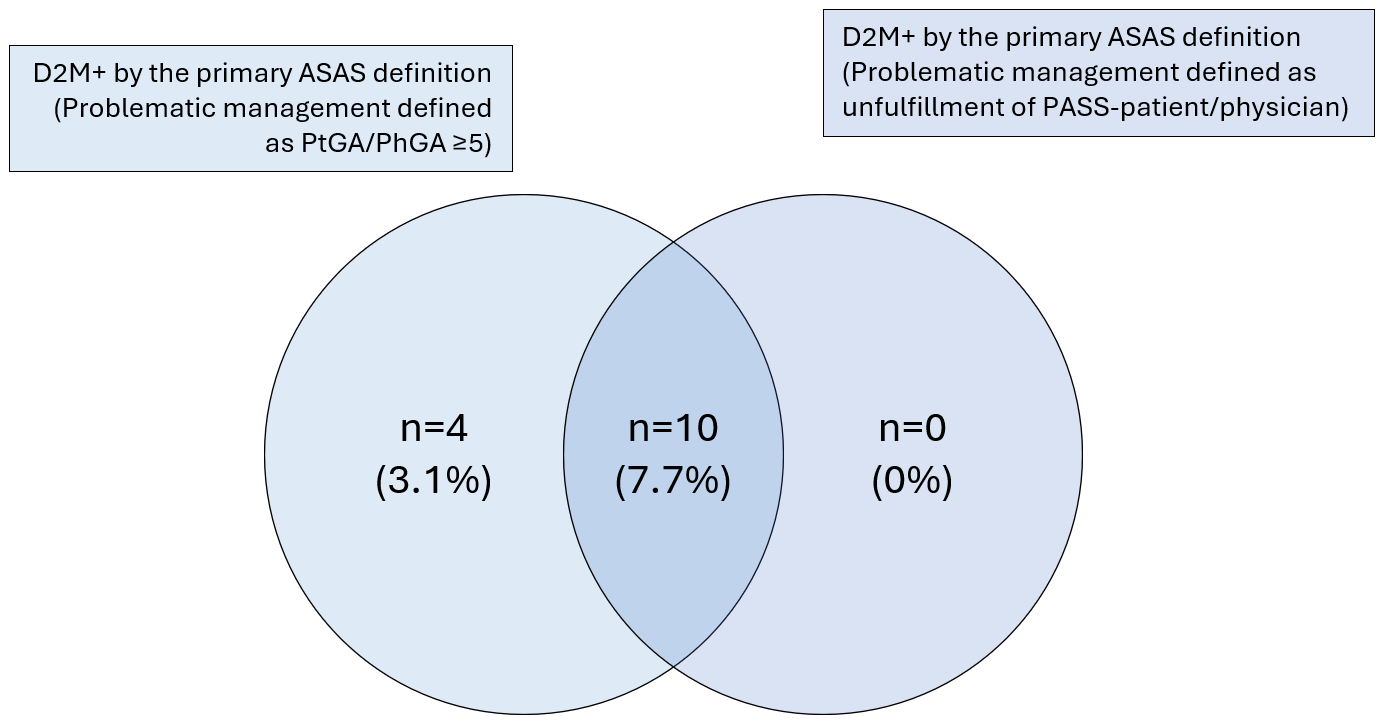


***Supplementary Figure S3.*** Sensitivity analysis: Comparison of the number of patients fulfilling the primary ASAS definition of D2M axSpA by means of the PtGA/PhGA vs. by means of the PASS-patient/physician (n=130).

ASAS: Assessment of SpondyloArthritis international Society; axSpA: axial spondyloarthritis; D2M: difficult-to-manage; PASS: patient/physician acceptable symptom state; PhGA: physician global assessment; PtGA: patient global assessment.

***Supplementary Table S7.*** Sensitivity analysis: Comparison of clinical characteristics between the patients fulfilling the primary ASAS definition of D2M axSpA only by means of the PtGA/PhGA vs. both by means of the PtGA/PhGA and PASS-patient/physician

| **Variable** | **Definition** |  |
| --- | --- | --- |
|  | **Patients fulfilling the primary ASAS definition only by means of the PtGA/PhGA**  **n=4** | **Patients fulfilling the primary ASAS definition by means of both the PtGA/PhGA and PASS-patient/physician**  **n=10** |
| ASDAS | 2.8 (0.4) | 2.8 (0.4) |
| BASDAI, 0-10 | 6.1 (0.7) | 6.3 (1.3) |
| CRP, mg/L | 2.8 (3.5) | 1.8 (1.1) |
| CRP ≥5mg/L, n (%) | 1 (25.0) | 0 (0.0) |
| Back pain VAS, 0-10 | 6.0 (0.8) | 7.1 (1.2) |
| ASAS-HI, 0-17 | 8.0 (2.4) | 9.1 (3.6) |
| SF-36 PCS, 0-100 | 37.8 (3.7) | 31.5 (5.4) |
| SF-36 MCS, 0-100 | 47.4 (14.8) | 40.0 (14.8) |
| Active peripheral manifestations^†^, n (%) | 0 (0.0) | 4 (44.4) |
| BSA ≥3%, n (%) | 0 (0.0) | 0 (0.0) |
| All values presented as mean (SD), unless otherwise indicated.  ^†^ Peripheral manifestations included arthritis, enthesitis and dactylitis.  Number of patients with a missing value: Active peripheral manifestations (n=1), BSA (n=1).  ASAS: Assessment of SpondyloArthritis international Society; ASAS-HI: ASAS-Health Index; ASDAS: Axial Spondyloarthritis Disease Activity Score; axSpA: axial spondyloarthritis; BASDAI: Bath Ankylosing Spondylitis Disease Activity Index; BSA: psoriasis body surface area; CRP: C-reactive protein; D2M: difficult-to-manage; PASS: patient/physician acceptable symptom state; PhGA: physician global assessment; PtGA: patient global assessment; SF-36 MCS: 36-Item Short Form Health Survey Mental Component Summary; SF-36 PCS: SF-36 Physical Component Summary; VAS: visual analogue scale. | | |
